# Low Cycle Fatigue Properties of Sc-Modified AA2519-T62 Extrusion

**DOI:** 10.3390/ma13010220

**Published:** 2020-01-04

**Authors:** Robert Kosturek, Lucjan Śnieżek, Janusz Torzewski, Marcin Wachowski

**Affiliations:** Faculty of Mechanical Engineering, Military University of Technology, 2 gen. S. Kaliskiego str., 00-908 Warsaw, Poland; lucjan.sniezek@wat.edu.pl (L.Ś.); janusz.torzewski@wat.edu.pl (J.T.); marcin.wachowski@wat.edu.pl (M.W.)

**Keywords:** AA2519, aluminum alloy, extrusion, mechanical properties, fatigue, fracture

## Abstract

This investigation presents the results of research on low cycle fatigue properties of Sc-modified AA2519-T62 extrusion. The basic mechanical properties of the investigated alloy have been established in the tensile test. The low cycle fatigue testing has been performed on five different levels of total strain amplitude: 0.4%; 0.5%; 0.6%; 0.7% and 0.8% with cycle asymmetry coefficient R = 0.1. For each level of total strain amplitude, the graphs of variations in stress amplitude and plastic strain amplitude in the number of cycles have been presented. The obtained results allowed to establish Ramberg-Osgood and Manson-Coffin-Basquin relationships. The established values of the cyclic strength coefficient and cyclic strain hardening exponent equal to k’ = 1518.1 MPa and n’ = 0.1702. Based on the Manscon-Coffin-Basquin equation, the values of the following parameters have been established: the fatigue strength coefficient σ’_f_ = 1489.8 MPa, the fatigue strength exponent b = −0.157, the fatigue ductility coefficient ε’_f_ = 0.4931 and the fatigue ductility exponent c = −1.01. The fatigue surfaces of samples tested on 0.4%, 0.6% and 0.8% of total strain amplitude have been subjected to scanning electron microscopy observations. The scanning electron microscopy observations of the fatigue surfaces revealed the presence of cracks in striations in the surrounding area with a high concentration of precipitates. It has been observed that larger Al_2_Cu precipitates exhibit a higher tendency to fracture than smaller precipitates having a higher concentration of scandium and zirconium.

## 1. Introduction 

The continual development of aluminum alloy has provided numerous light materials for engineering applications for over a century. High-strength aluminum alloys have found their application mainly in aerospace and automotive industry, where high specific strength allows to minimalize weight of construction [1,2,3]. Although the strongest of aluminum alloys owe their properties to precipitation hardening process (2xxx and 7xxx series), in recent years, a lot of effort has been undertaken to improve their parameters even further by different means [4,5]. These processes include operations as cryogenic rolling, equal channel angular pressing (ECAP), high-pressure torsion (HPT), accumulative extrusion bonding (AEB) and hydrostatic extrusion (HE) [6,7,8,9,10,11,12,13,14]. All operations except cryogenic rolling correspond to severe plastic deformation (SPD) process, in which deformation of material produces fine microstructure, improving its mechanical properties according to the Hall-Petch relationship [6,10,15]. In the case of cryogenic rolling plastic, deformation also causes grain refinement, but the additional factor is cryogenic temperature inhibiting grain recovery [13,14]. Another approach allowing to improve properties of aluminum alloys is the modification by alloying elements such as scandium, zirconium or hafnium [16,17,18,19,20,21]. Addition of scandium improves several properties of aluminum alloy by formation of Al_3_Sc phase in its microstructure [17,19]. This phase is characterized by lattice parameter (0.41 nm) close to the aluminum lattice (0.40494 nm), which in consequence entails high coherency of structures [22]. The influence of Al_3_Sc phase on properties of aluminum alloy includes grain refinement, dispersion hardening and increase of temperature needed for grain growth and recrystallization processes [19]. However, modifying aluminum with scandium creates some problems, for most effective modifying effect is achieved at high scandium concentrations (above 0.5%), which makes Al-Sc alloys very expensive due to the significant cost of scandium [16]. The solution to this problem is adding zirconium, which dissolves in Al_3_Sc phase replacing scandium atoms as the resulting Al_3_(Sc_1−*x*_, Zr*_x_*) intermetallic compound is formed [16]. The new phase is characterized by a large number of advantages compared to Al_3_Sc phase, such as lower tendency to coagulation at high temperature, which allows to maintain the capacity to hamper recrystallization and strengthen the alloy in a wider temperature range [16,19]. Although the Sc-modified aluminum alloys are the subject of much research, the main part of investigations is focused on microstructure and basic mechanical properties [23,24,25,26]. Some of the most important properties in terms of application in industry, especially in automotive and aerospace constructions, are fatigue properties since operating elements undergo cyclic loading during utilization [27,28,29]. Particularly, the low cycle fatigue behavior, which is characterized by the presence of high strain amplitude, is the crucial factor to estimate the cumulative damage and changes of material properties in its life cycle [28,30,31,32].

In this paper, the subject of the investigation is AA2519 alloy modified by scandium addition, which has been developed by the Institute of Non-Ferrous Metals, Light Metals Division in Skawina (Poland). This specific alloy is currently the object of a series of investigations performed by authors in terms of joining by explosive welding and friction stir welding [33,34,35,36]. AA2519 is a heat treatable aluminum-copper alloy with copper content within the 5.3% to 6.4% range, mainly used for military applications (e.g. advanced amphibious assault vehicles) due to its good ballistic properties [37]. The high specific strength of this alloy is the result of heat treatment in the form of precipitation hardening, which is realized in two steps: the solution treatment (annealing in 530 °C/2 h and cooling in cold water) and artificial aging (165 °C/10 h) [38,39]. After this process, the alloy is strengthened by θ′ precipitates, semi-coherent metastable Al_2_Cu phase with body-centered tetragonal crystal structure [38].

In terms of fatigue behavior, a substantial amount of investigations have been dedicated to aluminum–copper alloys (2xxx series) in recent years, mostly because of their wide use in the aircraft industry [3,27,28,31,40,41,42,43,44,45]. In this scientific literature, there are also investigations concerned with AA2519 and AA2219, which is the precursor of AA2519 [31,43,46]. Mohamed et al. revealed that low cycle fatigue behavior of AA2024 is influenced by the size of grains—the alloy with the grain size of 90 μm exhibited lower saturation stress and longer plateau in cyclic stress strain curve than alloy with the grain size of 150 μm [44]. Pec et al. reported that fracture area of 2024-T351 is characterized by the ductile mechanism with two size levels of dimples, which is the result of the presence of large inclusions and fine Al_2_Cu precipitates in the alloy microstructure [47]. Sharma et al. investigated low cycle fatigue behavior of AA2219-T87 providing very detailed data of fatigue parameters and description of material properties [31]. Some of their conclusions are that AA2219-T87 exhibits cyclic softening till failure at strain amplitudes between 0.4% and 1.5% and that the crack propagation mechanism depends on strain amplitude—for low strain is by crystallographic nature and for high strain by transgranular ductile striation [31]. Owolabi et al. reported that the fracture surface of AA2519-T8 depicted higher resistance to fatigue cracks nucleation and propagation compared to AA2219-T8, and the failure mechanism of AA2519 has dual character (brittle and ductile) with predominance of ductile fracture [43]. Baek et al. discovered that addition of 0.1% Sc to AA2519 results in higher resistance against fatigue crack nucleation due to presence of Al_3_(Sc,Zr) precipitates and fine subgrain structure [46]. Nowadays, the fatigue properties of Sc-modified 2xxx alloys are still a gap in the current state of the art, but undoubtedly, this gap will be gradually filled together with further development of light alloys in the years to come. In this paper, the low cycle fatigue properties of Sc-modified AA2519-T62 extrusion have been put under investigation.

## 2. Materials and Methods

The subject of the investigation was 5 mm thick AA2519 alloy extrusion with the chemical composition presented in Table 1.

**Figure 1:**
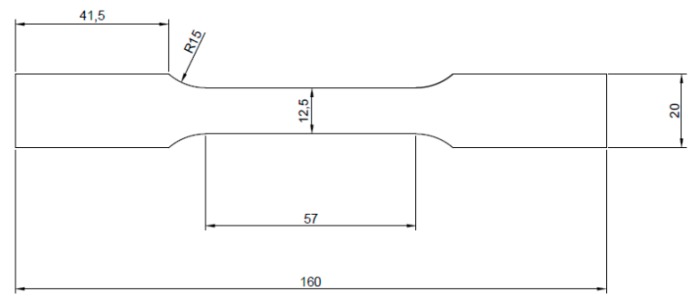
Scheme of sample for tensile testing. All dimensions are in mm. Thickness is equal to 5 mm.

**Figure 2:**
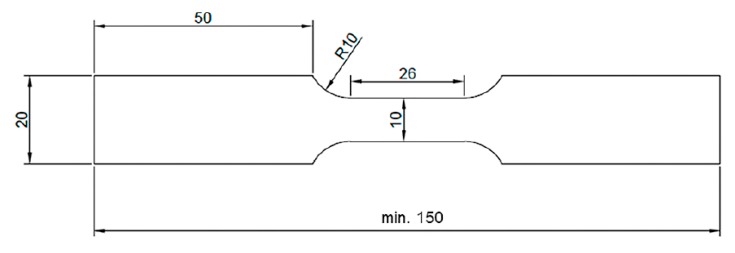
Scheme of sample for fatigue testing. All dimensions are in mm. Thickness is equal to 5 mm.

The alloy has been subjected to a two-step heat treatment: solution treatment (530 °C/2 h and cooling in cold water) and aging (165 °C/10 h). After the heat treatment, the metallurgical examinations and hardness measurements were carried out. As the part of metallographic sample preparation, a sample was cut from the extrusion using a precision diamond saw and then mounted in resin, grinded with abrasive paper of 80, 320, 600, 1200 and 2400 gradations, and polished using diamond pastes (3 and 1 μm gradation). The sample was etched by using Keller reagent (20 mL H_2_O + 5 mL HNO_3_ + 2 mL HF + 1 mL HCl) with etching time equal to 5 s. The Vickers microhardness was measured on the cross-section of polished sample by applying load of 0.98 N according with EN ISO 6507 standard. In order to perform the tensile and low cycle fatigue testing the samples have been prepared with the geometry presented in Figure 1 and Figure 2, respectively. The samples have been cut and examined in the direction parallel to the extrusion direction.

Tensile test was carried out on Instron 8802 MTL universal testing machine with WaveMatrix computer software in accordance with PN-EN ISO 6892 standard. The strain extensometer with a gauge length of 50 mm was used to measure deformation. During the test, the values of load, position and strain were recorded. Three samples have been examined, and the representative one has been presented in this investigation. Fatigue testing was carried out on Instron 8802 Servohydraulic Fatigue Testing System in accordance with ASTM E606/E606M standard. The strain during testing has been measured using a 2520-603 dynamic extensometer. Tests were performed on five different levels of total strain amplitude: 0.4%, 0.5%, 0.6%, 0.7% and 0.8% with cycle asymmetry coefficient R = 0.1. For each level of total strain amplitude, the three samples have been examined. The fatigue surfaces of samples tested on 0.4%, 0.6% and 0.8% have been subjected to scanning electron microscopy observations on Jeol JSM-6610 equipped in energy-dispersive x-ray spectroscopy (EDX) detector (Military University of Technology, Warsaw, Poland).

## 3. Results and Discussion

**Figure 3:**
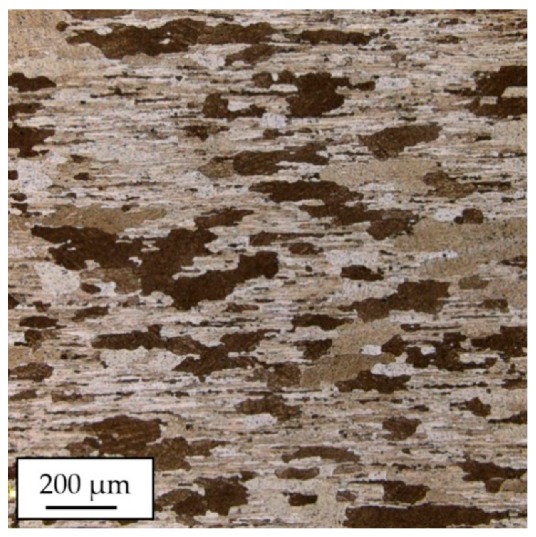
The microstructure of AA2519-T62 extrusion.

The microstructure of the investigated alloy is presented in Figure 3. Despite the fact that the examined microstructure reveals some differences in grain size, the measured microhardness is characterized by a small dispersion of values, and it equals 135.3 ± 5.9 HV0.1.

**Figure 4:**
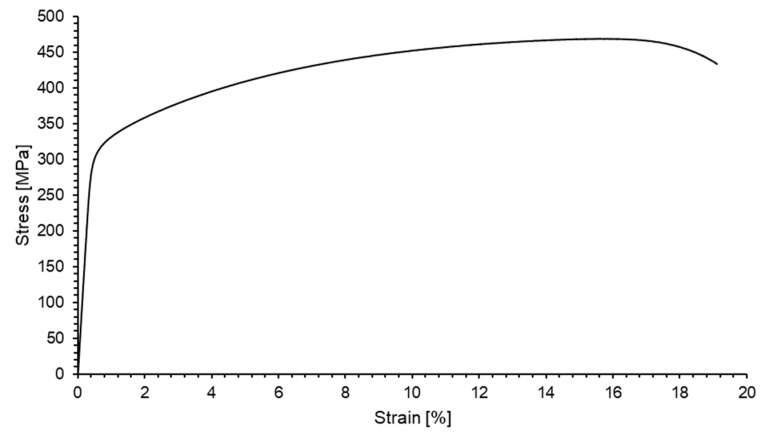
Tensile curve of AA2519-T62.

The tensile curve of the material is presented in Figure 4 with the obtained mechanical properties set in Table 2.

**Figure 5:**
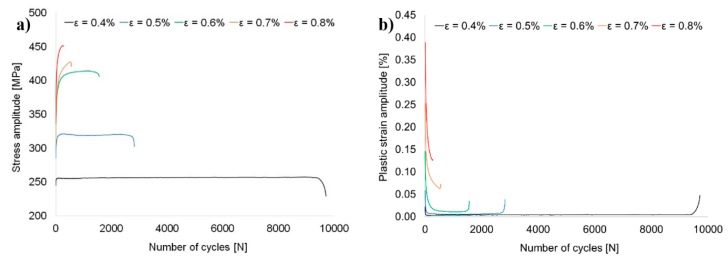
Variation of stress amplitude (**a**) and plastic strain amplitude (**b**) with the number of cycles.

The variations of stress amplitude and plastic strain amplitude with the number of cycles are presented in Figure 5a,b, respectively. It can be observed that the cyclic stress amplitude increases with an increase in the strain amplitude, whereas the fatigue life of the investigated alloy decreases with an increase of total strain amplitude. The analysis of these curves allows to draw the conclusion that for strain amplitudes equal to ε = 0.4% and ε = 0.5%, the samples are characterized by three stages of cyclic life: very short period of cyclic hardening (up to 200–300 cycles), cyclic stabilization and a final rapid drop in stress amplitude value until failure. In the case of the sample tested on ε = 0.6%, the stage of cyclic stabilization is unnoticeable, and the only stages to occur are cyclic hardening and cyclic softening before failure. As for the samples tested on higher levels of strain amplitude, ε = 0.7% and ε = 0.8%, the period of cyclic softening gradually disappears, and it can be observed that the sample ε = 0.8% undergoes cyclic hardening until failure.

**Figure 6:**
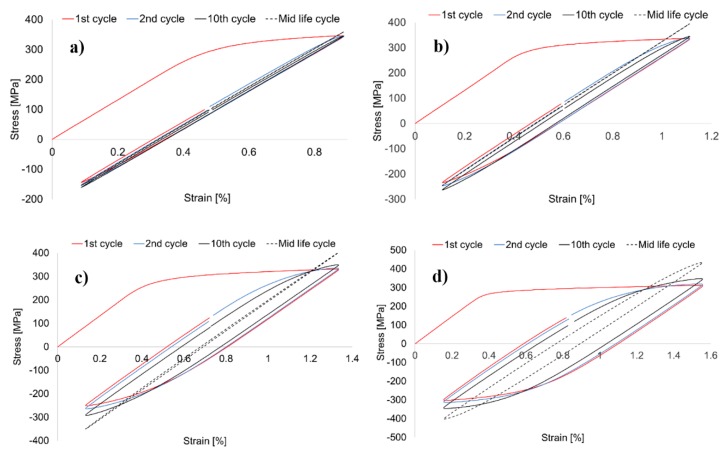

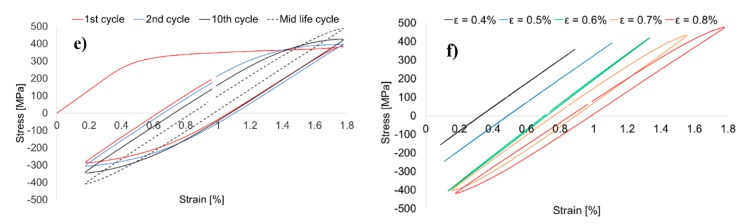
Stress-strain loops of sample tested with ε = 0.4% (**a**), ε = 0.5% (**b**), ε = 0.6% (**c**), ε = 0.7% (**d**), ε = 0.8% (**e**), and the comparison of mid-life loops of the samples (**f**).

The hysteresis stress-strain loops of analyzed material for various levels of strain amplitude are presented in Figure 6a–e. The stabilized, mid-life cycle loops for different strain amplitudes are compared in Figure 6f. In all analyzed samples, the first cycles have lower values of both tensile and compressive stress comparing to the stabilized, mid-life cycle loops, which confirms that material undergoes hardening during the first stage of its fatigue life.

**Figure 7:**
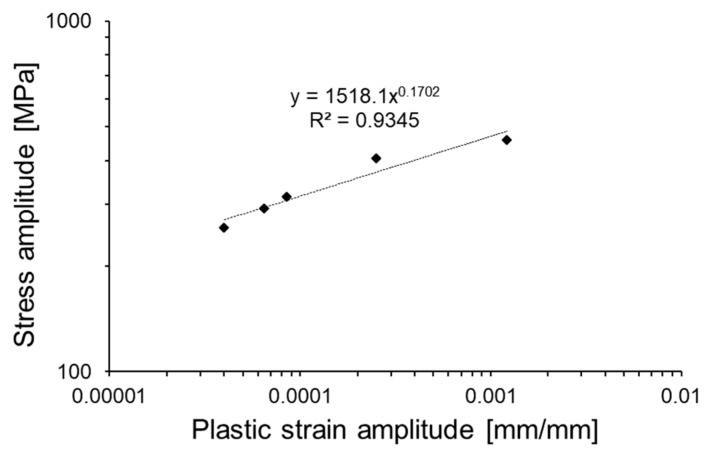
Stress amplitude versus plastic strain amplitude of stabilized hysteresis loops in log–log coordinates.

In the investigation performed by Sharma et al., authors reported that hysteresis loops of AA2219-T87 are inflected up to the 15th cycle as the result of interactions between moving dislocations and precipitates in the matrix [31]. In case of AA2519-T62, the strengthening phases are also coherent θ′′ and semicoherent θ′, but no inflected loops are observed as can be seen in Figure 6a–e. The stabilized hysteresis loops allowed to establish the values of stress and plastic strain amplitudes. The plot of stress versus plastic strain in log-log coordinates is presented in Figure 7.

The obtained curve can be described by the power function [36]:

(1) $$\sigma_{a}=k^{\prime }{\left(\varepsilon_{p}\right)}^{n^{\prime }}$$

where σ_a_ is the stress amplitude [MPa], ε_p_ is the plastic strain amplitude [mm/mm], *k*’ is the cyclic strength coefficient [MPa], and *n*’ is the cyclic strain hardening exponent. The values of k’ and n’ are established directly from function describing plot in Figure 7:

(2) $$\sigma_{a}=1518.1{\left(\varepsilon_{p}\right)}^{0.1702}$$

**Figure 8:**
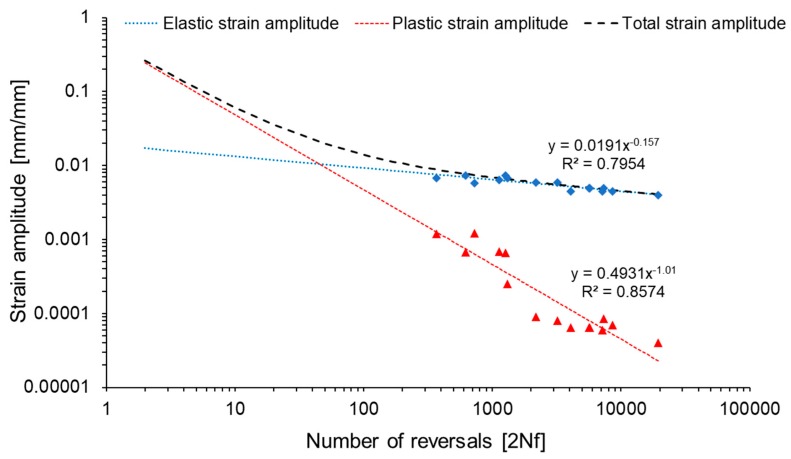
Number of half-cycle reversals vs strain amplitude in log–log coordinates.

The data from stabilized loops together with the number of reversals to failure allowed to describe low cycle fatigue properties of the material by Manson-Coffin-Basquin relationship. The equation allows to describe the total strain amplitude as the superposition of two functions: elastic strain amplitude and plastic strain amplitude vs the number of cycles. The obtained plot is presented in Figure 8.

The Manson–Coffin–Basquin equation is described by the following formula [36]:

(3) $$\varepsilon =\varepsilon_{e}+\;\varepsilon_{p}=\;\frac{\sigma_{f}^{\prime }}{E}{\left(2N_{f}\right)}^{b}+\;{\varepsilon^{\prime }}_{f}{\left(2N_{f}\right)}^{c}$$

where ε is the total strain amplitude [mm/mm], ε_e_ is the elastic strain amplitude [mm/mm], ε_p_ is the plastic strain amplitude [mm/mm], σ’_f_ is the fatigue strength coefficient [MPa], E is the Young modulus [MPa], b is the fatigue strength exponent, ε’_f_ is the fatigue ductility coefficient and c is the fatigue ductility exponent. The values of defined parameters are established from functions describing the plots in Figure 8:

(4) $$\varepsilon =\varepsilon_{e}+\;\varepsilon_{p}=\;\frac{1489.8}{78000}{\left(2N_{f}\right)}^{-0.157}+\;0.4931\;{\left(2N_{f}\right)}^{-1.01}$$

**Figure 9:**
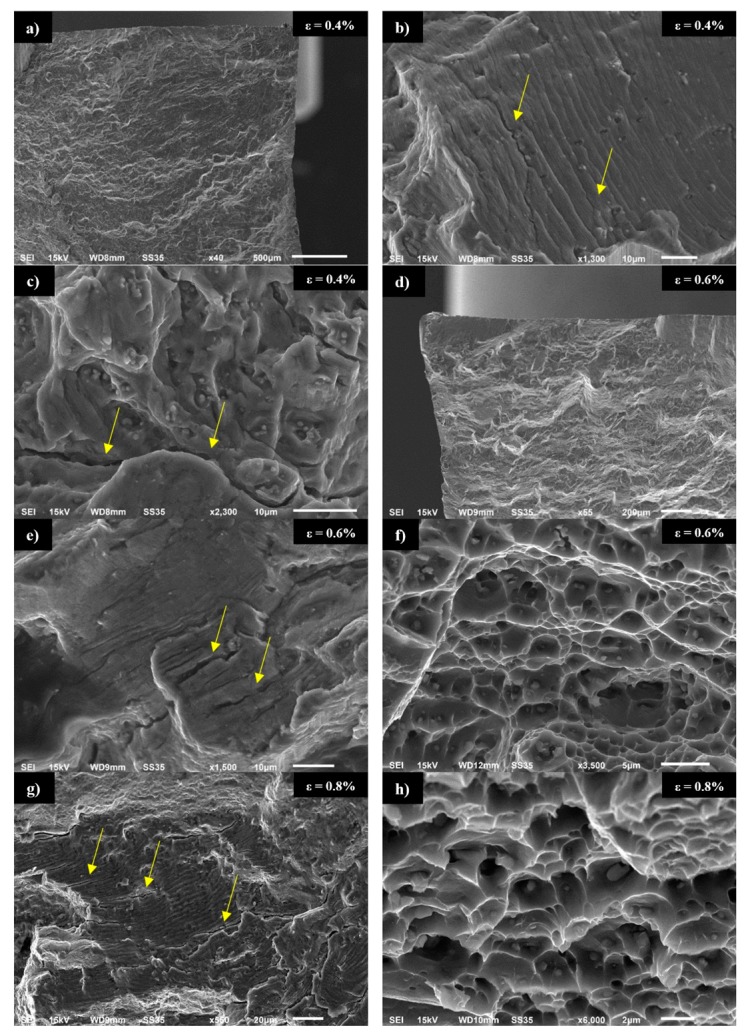
Scanning electron microscopy images of fatigue surface: initiation zone for ε = 0.4% (**a**), fatigue striations for ε = 0.4% (**b**), dimple structure for ε = 0.4% (**c**), initiation zone for ε = 0.6% (**d**), fatigue striations for ε = 0.6% (**e**), dimple structure for ε = 0.6% (**f**), fatigue striations for ε = 0.8% (**g**), dimple structure for ε = 0.8% (**h**).

The fatigue surfaces of samples tested on 0.4%, 0.6% and 0.8% of total strain amplitude have been subjected to scanning electron microscopy observations. The initiation zone of sample tested with ε = 0.4% can be observed in Figure 9a. The fracture surface is characterized by a mixed ductile and brittle fracture with the predominance of ductile fracture. As it can be seen in Figure 9b, the local presence of fatigue striations has been reported with local occurrence of cracks localized in the surrounding of precipitates (marked with yellow arrows). This type of cracks indicates the local acceleration of material decohesion caused by stress concentration on the precipitates. In Figure 9c, the characteristic dimple structure can be observed. The dimples are the effect of significant plastic deformation in the surroundings of non-coherent, large Al_2_Cu precipitates. It is also noticed that areas of large precipitate concentration promote occurrence of cracks (marked with yellow arrows). The fatigue surface of the sample tested with ε = 0.6% presented in Figure 9d is also characterized by a mixed type of fracture, but in this case, the participation of ductile fracture is higher than in the previous sample what is a direct result of the strain amplitude increase. The consequences of a higher degree of plastic deformation are possible to observe in Figure 9e, where numerous, large cracks occur in fatigue striations (marked with yellow arrows). Figure 9f presents the dimple structure with the precipitates localized on the bottom of each dimple.

**Figure 10:**
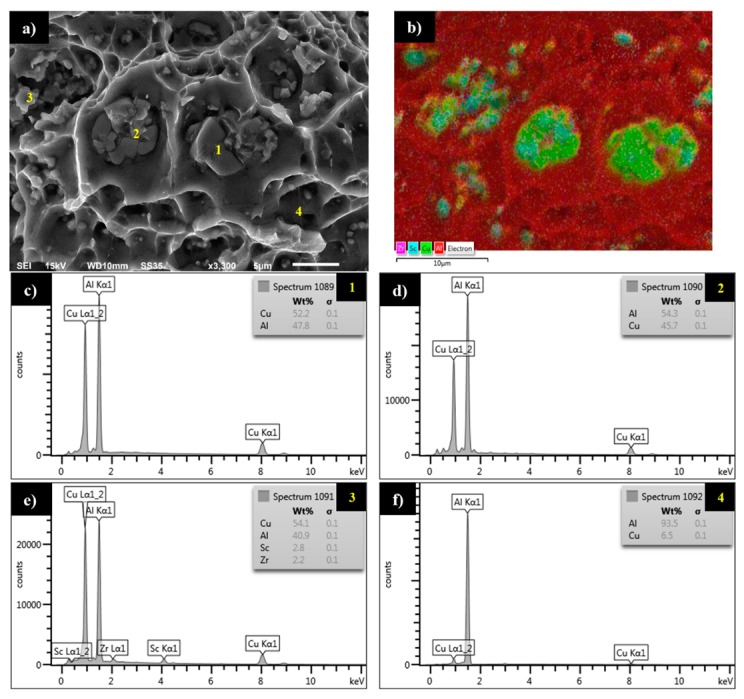
Scanning electron microscopy images of fatigue surface (**a**) together with element distributions on the surface of sample (**b**) and results of EDX analysis in yellow-marked points 1 to 4 (**c**–**f**).

For the sample tested with ε = 0.8%, the striations and dimples are presented in Figure 9g,h, respectively. The comparison of these images with images presented in Figure 9e,f allows to conclude that together with increasing strain amplitude, the participation of cracks in striations escalate, and the edges of dimple structure become sharper, indicating a more ductile character of fatigue decohesion. Additionally, based on observations of fatigue surface for the sample tested with ε = 0.8%, it has been reported that it is possible to distinguish two main types of precipitates. In Figure 10a, it can be observed that larger precipitates with size within the range 5–10 μm tend to fracture during cyclic loading. The second type of precipitate is slightly smaller with a size of about 1 μm. The results of the analysis of element distribution on the surface of the sample presented in Figure 10b show that smaller precipitates are characterized by a higher concentration of scandium. This phenomenon has been confirmed by the results of EDX analysis presented in Figure 10c–f. The larger precipitates (marked with yellow points 1 and 2) with high participation of cracks correspond to Al_2_Cu equilibrium phase according to spectrums in Figure 10c,d. At the same time EDX analysis of smaller precipitates (yellow point 3), presented in Figure 10e, indicates an elevated concentration of scandium (2.8%) and zirconium (2.2%). Figure 10f shows the spectrum of the alloy matrix.

## 4. Conclusions

The performed research on low cycle fatigue properties of Sc-modified AA2519-T62 extrusion allowed the following conclusions to be drawn:

1. The investigated material has three stages of cyclic life: a very short period of cyclic hardening, cyclic stabilization and a final rapid drop in stress amplitude value until failure. Together with increasing strain amplitude, the stage of cyclic stabilization disappears, and for the highest value of amplitude (0.8%), it is impossible to distinguish.
2. The obtained results allowed to establish Ramberg-Osgood and Manson-Coffin-Basquin relationships. The established values of the cyclic strength coefficient and cyclic strain hardening exponent equal to k’ = 1518.1 MPa and n’ = 0.1702. For the Manscon-Coffin-Basquin equation, the values of the following parameters have been established: the fatigue strength coefficient σ’f = 1489.8 MPa, the fatigue strength exponent b = −0.157, the fatigue ductility coefficient ε’f = 0.4931 and the fatigue ductility exponent c = −1.01.
3. The scanning electron microscopy observations of the fractures revealed the presence of cracks in striations in the surrounding area with a high concentration of precipitates. Additionally, it has been reported that larger Al_2_Cu precipitates exhibit a higher tendency to fracture than smaller precipitates having an elevated concentration of scandium and zirconium.

## Figures and Tables

**Table 1:** Chemical composition of AA2519-T62 extrusion.

Fe	Si	Cu	Zn	Ti	Mn	Mg	Ni	Zr	Sc	V	Al
0.11	0.08	6.32	0.05	0.08	0.17	0.33	0.02	0.19	0.16	0.10	Base

**Table 2:** Mechanical properties of Sc-modified AA2519-T62 extrusion.

Young Modulus (E)	Yield Strength (R_e0,2_)	Tensile Strength (R_m_)	Fracture Stress (R_u_)	Elongation (A)
78 GPa	312 MPa	469 MPa	434 MPa	19%
